# Association between dietary antioxidants, serum albumin/globulin ratio and quality of life in esophageal squamous cell carcinoma patients: a 7-year follow-up study

**DOI:** 10.3389/fonc.2025.1428214

**Published:** 2025-01-23

**Authors:** Juwei Zhang, Jinsong Zhou, Siting Chen, Yue Huang, Zheng Lin, Yuan Deng, Minglian Qiu, Zhisheng Xiang, Zhijian Hu

**Affiliations:** ^1^ Department of Epidemiology and Health Statistics, Fujian Provincial Key Laboratory of Environment Factors and Cancer, School of Public Health, Fujian Medical University, Fuzhou, China; ^2^ Department of Thoracic Surgery, The First Affiliated Hospital of Fujian Medical University, Fuzhou, China; ^3^ Department of Epidemiology, Clinical Oncology School of Fujian Medical University, Fujian Cancer Hospital, Fuzhou, China; ^4^ Department of Key Laboratory of the Ministry of Education for Gastrointestinal Cancer, Fujian Medical University, Fuzhou, China

**Keywords:** esophageal squamous cell carcinoma, albumin-to-globulin ratio, dietary antioxidant index, health-related quality of life, time to deterioration model

## Abstract

**Objective:**

This study aimed to explore the association between dietary antioxidant index (DAI) combined with serum albumin-to-globulin ratio (AGR) and postoperative Health-related quality of life (HRQOL) in patients with esophageal squamous cell carcinoma (ESCC).

**Methods:**

All patients were newly diagnosed with ESCC and underwent radical esophagectomy. Dietary data and routine blood tests were collected preoperatively to compute DAI and AGR. HRQOL was assessed over 7 years post-surgery via telephone follow-up and analyzed longitudinally using a time to deterioration (TTD) model. The deterioration times were compared using the log-rank test, and the association of the combined DAI and AGR index with postoperative quality of life in ESCC patients was examined through Cox regression models.

**Results:**

A total of 238 ESCC patients were included in the study. The results indicate that compared to the low DAI-AGR group, the high DAI-AGR group had a lower rate of deterioration events, and the time to deterioration in emotional functioning (p=0.014), dysphagia (p=0.042), and speech problems (p=0.023) were significantly delayed. Cox proportional hazard model revealed that preoperative high DAI-AGR was associated with improvement in emotional functioning (HR=0.575, 95% CI: 0.368-0.898) and speech problems (HR=0.525, 95% CI: 0.298-0.925) in ESCC patients postoperatively, which remained significant even after adjusting for covariates. The stratified analysis revealed that this improvement was associated with demographic and clinical characteristics.

**Conclusions:**

Our findings suggest that high preoperative DAI-AGR is linked to enhanced postoperative HRQOL in ESCC patients, offering crucial insights for patients, practitioners, and researchers.

## Introduction

Esophageal cancer (EC) is the 11th most common cancer worldwide and the seventh leading cause of cancer-related deaths, with 511,000 new cases and 445,000 deaths reported in 2022 (1). Esophageal squamous cell carcinoma (ESCC) accounts for approximately 85% of EC cases, particularly in China, where its incidence in males is twice that in females (2). While advances in treatment have improved the survival rates of ESCC patients, treatment-related side effects significantly impact postoperative quality of life (QOL), a growing concern for both surgeons and patients (3). Health-related quality of life (HRQOL) has become a critical endpoint in medical research, encompassing physical, psychological, and social dimensions of health (4). Standardized tools, such as the EORTC QLQ-C30 (5) and its esophageal cancer-specific supplement OES-18 (6), are widely used to assess QOL in ESCC patients, enabling the identification of factors influencing postoperative outcomes and guiding strategies to improve their well-being.

Malnutrition and inflammation are associated with poor prognosis in upper gastrointestinal (UGI) cancer patients (7). Serum protein components, including albumin (ALB) and globulin (GLB), are widely used to evaluate the nutritional status and disease severity of cancer patients (8). Epidemiological evidence suggests that low ALB or high GLB levels are often associated with increased mortality and recurrence rates in various cancers (9). However, ALB and GLB levels are influenced by confounding factors, limiting their precision in reflecting nutritional and inflammatory status (10). To address this, we introduced the albumin-to-globulin ratio (AGR), which our previous research has shown to be positively correlated with the quality of life in patients with ESCC (11). Nevertheless, the impact of dietary factors on nutritional status was not considered. Antioxidant components in the diet are crucial for esophageal cancer treatment and prognosis, with evidence suggesting that vitamin C supplementation can help regulate inflammation and carcinogenesis (12). Additionally, immune-enhancing diets rich in antioxidants have been shown to reduce immune deterioration caused by neoadjuvant therapy in esophageal cancer patients and improve postoperative quality of life (13), findings that align with similar research in other cancers (14). To better evaluate the role of dietary antioxidants in the preoperative nutritional status and its impact on postoperative quality of life in ESCC patients, this study employs the dietary antioxidant index (DAI) (15) to assess overall dietary antioxidant capacity, moving beyond the focus on single-antioxidant foods or nutrients.

The DAI reflects the inflammatory potential of the diet, while the AGR is an important marker of the body’s nutritional and inflammatory status (15, 16). Both indices may influence HRQOL through shared inflammatory pathways or metabolic regulation. However, most existing studies have focused separately on the effects of DAI or AGR on cancer prognosis, with limited research examining their combined impact. Additionally, ESCC has unique pathological and clinical characteristics (17), yet studies investigating factors affecting HRQOL in ESCC patients, particularly comprehensive indices, remain scarce. Investigating the combined effects of DAI and AGR could elucidate their distinct mechanisms influencing HRQOL in ESCC patients and provide a basis for future nutritional and inflammatory interventions. Furthermore, uncovering their impact on HRQOL may promote the development of personalized nutritional guidance and inflammation management strategies, ultimately improving patient quality of life and survival rates.

Hence, we collected preoperative dietary information and biochemical indicators of ESCC patients. The quality of life of ESCC patients was followed up for 7 years to explore the impact of preoperative AGR and DAI combined indicators on their quality of life. To our knowledge, this study is the first to combine two indicators, aiming to provide new insights into the improvement of postoperative quality of life in patients with ESCC.

## Materials & methods

### Study design and participants

All included patients were (1) newly diagnosed esophageal squamous cell carcinoma patients at Fujian Cancer Hospital and the First Hospital of Fujian Medical University from December 2014 to November 2021; (2) Having undergone radical esophagectomy; (3) Postoperative pathological diagnosis of esophageal squamous cell carcinoma; (4) Clear TNM staging; and (5) no history of radio-chemotherapy before surgery. The exclusion criteria include patients with other tumors, patients with metastatic tumors or recurrent esophageal cancer, and patients with incomplete clinical case data. Committee on Cancer Tumor Lymph Node Metastasis (TNM) classification system. This study received approval (approval number: 201495) from the Ethics Committee of Fujian Medical University, and all participants gave informed consent before participating in the research.

### Demographics and clinical characteristics

Demographic and clinical data, including age, gender, radio-chemotherapy, and TNM stage, were extracted from electronic medical records (EMR). Albumin and globulin counts were assessed through blood routine indexes one week before surgery, and the ratios were computed. Subsequently, the ESCC patients were stratified into high and low groups based on the median (AGR=1.428).

### Questionnaire assessment

All participants in the study underwent face-to-face interviews conducted by proficient interviewers using a questionnaire designed specifically for this study. This questionnaire encompassed a range of information, including general details (age, gender, ethnicity, education level, place of residence, marital status, etc.), lifestyle habits (smoking history, alcohol consumption, tea consumption, etc.), family history of tumors, past medical conditions, occupational history, and more.

To examine the long-term food consumption patterns and dietary habits of the participants, a semi-quantitative food frequency questionnaire (FFQ) was employed (18). This questionnaire assessed the frequency and intake of diverse foods over the past year. It comprised a total of 185 food items distributed across 12 categories, encompassing staple foods, potatoes, eggs, soy products, animal meat, fish, shrimp, crab and shellfish, vegetables, fruits, and dairy. To assess the intake of each food item, information on the frequency of consumption per day, week, month, or year, along with the quantity of each intake and the cumulative duration of intake was collected. In cases where participants were unsure about the food weight, reference was made to the food model legend and food weight conversion table. The conversion of weekly food intake frequency involved transforming the frequency to “times/week” using an equivalence formula (e.g. if eggs were consumed once a day, 1 time/day × 7 days/week = 7 times/week). Subsequently, the weekly intake of each food was computed (weekly intake = weekly intake frequency × quantity consumed each time) in grams (g). Exclusion criteria were applied, including extreme daily intake (<1% or >99%) or extreme intake of any single food (>99.5%), as well as an intake of fewer than 10 types of food. Finally, the daily intakes of six antioxidants, namely vitamins A, C, and E, manganese, selenium, and zinc, were calculated based on the Chinese Food Composition Table (6th edition) using the nutrient composition of each food.

The standardized questionnaires employed in this study were the EORTC Quality of Life Questionnaire-Core Questionnaire (EORTC QLQ-C30, version 3.0) (5) and the Esophageal Cancer Module (EORTC QLQ-OES18) (6). The comprehensive content of the questionnaire has been elaborated in our prior publications (11). The above questionnaires were administered by trained interviewers within three days of the patient’s admission to collect comprehensive information about the study participants.

### Follow up

Patients who underwent esophagectomy were subject to monitoring for the assessment of their HRQOL. This monitoring encompassed follow-up assessments at 3-month intervals throughout the first year and 6-month intervals in the subsequent years. The follow-up was conducted via telephone by trained interviewers.

### Statistical methods

The Quality-of-Life Scale was assessed using the Time to Deterioration (TTD) model, which considered both the time to deterioration and the frequency of deterioration events in each domain. TTD model was developed using the QoLR package (19) within the R software to compute scores on the EORTC QLQ-C30/EORTC QLQ-OES18 scale. In this research, time to deterioration was defined as the time from the study commencement to the first occurrence of a 5-point decrease in the quality of life score compared to the baseline. If no deterioration occurred, the assessment was revisited at the time of the last completed quality of life evaluation (20).

The Dietary Antioxidant Index (DAI) for all participants was calculated using data collected from the Food Frequency Questionnaire (FFQ). The total antioxidant capacity of participants’ food intake was assessed using Wright’s recommended method (21). For each participant, DAI was calculated by summing the normalized consumption of six antioxidants (i), vitamins A, C, and E, manganese, selenium, and zinc, which were derived from food sources only (i.e., excluding dietary supplements). This is described in the formula below. In the equation, x_i_ is the daily intake of antioxidants; μ_i_ is the mean value of antioxidant I throughout the overall study; and S_i_ is the SD of μ. ESCC patients were stratified into two groups: a low group (DAI<-1.802) and a high group (DAI ≥ -1.802), with the median DAI serving as the dividing point.


$$\text{DAI}=\sum_{\text{i}=1}^{\text{n}=6}\frac{{\text{X}}_{\text{i}}-{\text{μ}}_{\text{i}}}{{\text{S}}_{\text{i}}}$$

The chi-square test was used to compare the distribution of categorical variables between the two groups. For quantitative variables exhibiting skewed distribution, the median (interquartile range (IQR)) was used for comparison. Furthermore, qualitative variables are presented using numbers (percentages). The log-rank test is applied to compare the time to deterioration, and univariate and multivariate Cox regression analyses are conducted to identify factors associated with the quality of life in patients with ESCC. The statistical analysis described above was conducted using SPSS version 22.0. Hazard ratios (HR) were estimated with a 95% confidence interval (95% CI). All statistical tests were two-sided, using a significance level of 5%, and a p-value less than 0.05 was deemed statistically significant.

## Results

### Baseline characteristics of the study population

A total of 238 patients with ESCC were included in this study. According to the grouping of AGR and DAI, groups with AGR≥1.428 and DAI≥-1.802 are classified as the high DAI-AGR group, while the remaining combinations are classified as the low DAI-AGR group. The chi-square test results showed that income was unevenly distributed between the two groups. The income of the high DAI-AGR group was significantly higher than that of the low DAI-AGR group (p<0.05), and the remaining baseline characteristics were evenly distributed between the two groups (p≥0.05), as shown in **Table 1**.

**Table 1:** Demographic and clinical characteristics of 238 patients with ESCC.

Variables	Low DAI-AGR [n (%)]	High DAI-AGR [n (%)]	*χ* ^2^	*P* value
Total	178 (74.8%)	60 (23.2%)		
Sex			1.586	0.208
Female	47 (26.4%)	11 (18.3%)		
Male	131 (73.6%)	49 (81.7%)		
Age(years)			0.138	0.710
<60	90 (50.6%)	32 (53.3%)		
≥60	88 (49.4%)	28 (46.7%)		
Marital status			2.935^*^	0.206
Unmarried	2 (1.1%)	0 (0.0%)		
Married	172 (96.6%)	56 (93.3%)		
Widowed	4 (2.2%)	4 (6.7%)		
Education level			2.528	0.112
Primary and below	121 (68.0%)	34 (56.7%)		
Junior high school and above	57 (32.0%)	26 (43.3%)		
Family income per month			15.312	<0.001
<2000	73 (41.0%)	8 (13.3%)		
≥2000	105 (59.0%)	52 (86.7%)		
Smoker			1.214	0.271
No	58 (32.6%)	15 (25.0%)		
Yes	120 (67.4%)	45 (75.0%)		
Drinker			2.018	0.155
No	96 (53.9%)	26 (43.3%)		
Yes	82 (46.1%)	34 (56.7%)		
Postoperative radio-chemotherapy			0.797	0.372
No	95 (53.4%)	36 (60.0%)		
Yes	83 (46.6%)	24 (40.0%)		
TNM stage			1.087	0.297
I–II	90 (50.6%)	35 (58.3%)		
III–IV	88 (49.4%)	25 (41.7%)		
ALT (U/L)			1.735	0.188
≤16	102 (57.3%)	28 (47.5%)		
>16	76 (42.7%)	31 (52.5%)		
AST (U/L)			0.171	0.679
≤20	88 (49.4%)	31 (52.5%)		
>20	90 (50.6%)	28 (47.5%)		
Creatinine (μmol/L)			0.119	0.730
≤75	87 (50.0%)	30 (52.6%)		
>75	87 (50.0%)	27 (47.4%)		

*Fisher’s exact test.

ALT, alanine aminotransferase; AST, aspartate aminotransferase.

Data are expressed as proportion n (%).

*P*-value less than 0.05 was considered significant.

*P*-value based on chi-square test and Fisher’s exact test.

### Baseline quality-of-life scores

The baseline quality of life score is described using the median and quartile. There was a significant difference in baseline physical functioning (p=0.014) and social functioning (p=0.004) between the two groups, while there was no statistical difference in other dimensions (**Table 2**).

**Table 2:** Preoperative EORTC scale baseline scores in post-ESCC patients.

	Baseline HRQOL scores [M (IQR)], n=238			
Domain/scale	Low DAI-AGR	High DAI-AGR	Z	*P* value
QLQ-C30				
Global health status/QOL	83.33 (66.67, 87.33)	66.67 (66.67. 83.33)	-1.686	0.092
Functional scales				
Physical functioning	100.00 (93.33, 100.00)	100.00 (86.67, 100.00)	-2.462	0.014
Role functioning	100.00 (100.00, 100.00)	100.00 (100.00, 100.00)	-0.348	0.728
Emotional functioning	91.67 (75.00, 100.00)	91.67 (75.00, 100.00)	-0.053	0.957
Cognitive functioning	100.00 (83.33, 100.00)	100.00 (100.00, 100.00)	-1.082	0.279
Social functioning	66.67 (66.67, 100.00)	100.00 (66.67, 100.00)	-2.898	0.004
Symptom scales				
Fatigue	0.00 (0.00, 22.22)	11.11 (0.00, 33.33)	-1.600	0.110
Nausea/vomiting	0.00 (0.00, 0.00)	0.00 (0.00, 0.00)	-0.290	0.771
Pain	0.00 (0.00, 16.67)	16.67 (0.00, 33.33)	-1.235	0.217
Dyspnea	0.00 (0.00, 0.00)	0.00 (0.00, 0.00)	-0.968	0.333
Insomnia	0.00 (0.00, 33.33)	0.00 (0.00, 33.33)	-1.399	0.162
Appetite loss	0.00 (0.00, 33.33)	0.00 (0.00, 33.33)	-0.525	0.600
Constipation	0.00 (0.00, 0.00)	0.00 (0.00, 0.00)	-1.617	0.106
Diarrhea	0.00 (0.00, 0.00)	0.00 (0.00, 0.00)	-0.428	0.669
QLQ-QES18				
General symptom scales				
Dysphagia	88.89 (66.67, 100.00)	77.78 (66.67, 100.00)	-0.581	0.561
Eating problems	0.00 (0.00, 16.67)	8.33 (0.00, 16.67)	-1.330	0.184
Reflux	0.00 (0.00, 0.00)	0.00 (0.00, 0.00)	-0.167	0.867
Odynophagia	11.11 (0.00, 22.22)	11.11 (0.00, 22.22)	-0.830	0.406
General symptom items				
Trouble swallowing saliva	0.00 (0.00, 0.00)	0.00 (0.00, 0.00)	-1.291	0.197
Choking when swallowing	16.67 (0.00, 33.33)	33.33 (0.00, 33.33)	-0.755	0.451
Dry mouth	0.00 (0.00, 0.00)	0.00 (0.00, 33.33)	-1.572	0.116
Trouble with taste	0.00 (0.00, 0.00)	0.00 (0.00, 0.00)	-0.142	0.887
Coughing	0.00 (0.00, 0.00)	0.00 (0.00, 0.00)	-0.767	0.443
Speech problems	0.00 (0.00, 0.00)	0.00 (0.00, 0.00)	-0.844	0.399

Data are expressed as median [interquartile range (IQR)].

*P*-value less than 0.05 was considered significant.

*P*-values were calculated using the Mann-Whitney U test.

### Association between preoperative DAI and AGR and postoperative quality of life deterioration rate and deterioration time in ESCC patients

Compared with the low DAI-AGR group, the high DAI-AGR group had a lower incidence of deterioration events, including emotional functioning (p<0.001), cognitive functioning (p=0.020), social functioning (p=0.007), pain (p=0.018), dyspnea (p=0.005), insomnia (p=0.035), appetite loss (p=0.011), dysphagia (p=0.016), eating problems (p=0.044), odynophagia (p=0.002), trouble swallowing saliva (p=0.011), choking when swallowing (p=0.003), trouble with taste (p=0.006), coughing (p=0.006) and speech problems (p<0.001) (**Table 3**). In addition, the results of log-rank test analysis show that the time to deterioration of emotional functioning (p=0.014), dysphagia (p=0.042), and speech problems (p=0.023) in patients with high DAI-AGR group was significantly delayed (**Table 4**).

**Table 3:** The incidence of TTD events in each dimension of the QLQ-C30/QLQ-OES18 scale in ESCC patients.

Domain/scale	Low DAI-AGR [n (%)]	High DAI-AGR [n (%)]	*χ^2^*	*P* value
QLQ-C30				
Global health status/QOL	139 (78.1%)	48 (80.0%)	0.097	0.755
Functional scales				
Physical functioning	144 (80.9%)	50 (83.3%)	0.176	0.674
Role functioning	128 (71.9%)	42 (70.0%)	0.080	0.777
Emotional functioning	122 (68.5%)	23 (38.3%)	17.198	<0.001
Cognitive functioning	102 (57.3%)	24 (40.0%)	5.393	0.020
Social functioning	118 (66.3%)	28 (46.7%)	7.289	0.007
Symptom scales				
Fatigue	128 (71.9%)	35 (58.3%)	3.833	0.050
Nausea/vomiting	119 (66.9%)	32 (53.3%)	3.537	0.060
Pain	114 (64.0%)	28 (46.7%)	5.631	0.018
Dyspnea	114 (64.0%)	26 (43.3%)	7.947	0.005
Insomnia	119 (66.9%)	31 (51.7%)	4.442	0.035
Appetite loss	119 (66.9%)	29 (48.3%)	6.546	0.011
Constipation	76 (42.7%)	20 (33.3%)	1.635	0.201
Diarrhea	109 (61.2%)	30 (50.0%)	2.332	0.127
QLQ-QES18				
General symptom scales				
Dysphagia	138 (77.5%)	37 (61.7%)	5.800	0.016
Eating problems	126 (70.8%)	34 (56.7%)	4.061	0.044
Reflux	147 (82.6%)	44 (73.3%)	2.423	0.120
Odynophagia	110 (61.8%)	23 (38.3%)	10.021	0.002
General symptom items				
Trouble swallowing saliva	87 (48.9%)	18 (30.0%)	6.486	0.011
Choking when swallowing	105 (59.0%)	22 (36.7%)	8.984	0.003
Dry mouth	93 (52.2%)	25 (41.7%)	2.010	0.156
Trouble with taste	77 (43.3%)	14 (25.2%)	7.544	0.006
Coughing	99 (55.6%)	21 (35.0%)	7.631	0.006
Speech problems	88 (49.4%)	14 (23.3%)	12.487	<0.001

Data are expressed as proportion n (%).

*P*-value less than 0.05 was considered significant.

*P*-value based on chi-square test.

**Table 4:** Determination of clinically meaningful time to deterioration in the EORTC QLQ -C30/EORTC QLQ -OES18 scale in ESCC patients.

	Time to deterioration [M (95%CI)], n=238			
Domain/scale	Low DAI-AGR	High DAI-AGR	*χ* ^2^	*P* value
QLQ-C30				
Global health status/QOL	12.945 (9.973-15.916)	11.992 (2.167-16.239)	1.651	0.199
Functional scales				
Physical functioning	12.945 (11.648-14.241)	14.357 (11.678-17.037)	0.166	0.684
Role functioning	16.559 (12.582-20.535)	18.103 (13.992-22.214)	0.589	0.443
Emotional functioning	23.458 (18.672-28.244)	64.493 (30.299-98.687)	6.083	0.014
Cognitive functioning	44.747 (31.514-57.980)	59.663 (12.653-106.674)	0.311	0.577
Social functioning	21.848 (16.397-27.299)	26.382 (18.349-34.415)	0.481	0.488
Symptom scales				
Fatigue	15.869 (13.163-18.574)	18.201 (9.800-26.603)	0.588	0.443
Nausea/vomiting	22.505 (17.141-27.869)	26.415 (13.547-39.282)	0.042	0.898
Pain	24.805 (15.245-34.365)	27.828 (21.894-33.761)	1.074	0.300
Dyspnea	23.359 (17.398-29.321)	40.378 (19.997-60.759)	2.147	0.143
Insomnia	24.674 (19.769-29.578)	31.671 (14.761-48.582)	0.546	0.460
Appetite loss	21.585 (16.347-26.824)	31.014 (15.596-46.433)	1.706	0.192
Constipation	49.018 (-)	50.497 (19.891-81.103)	0.032	0.858
Diarrhea	25.856 (20.636-31.077)	27.828 (15.633-40.022)	0.049	0.825
QLQ-QES18				
General symptom scales				
Dysphagia	12.977 (10.649-15.305)	17.018 (10.751-23.286)	4.137	0.042
Eating problems	15.901 (13.619-18.184)	21.815 (12.043-31.587)	0.754	0.385
Reflux	16.099 (13.881-18.316)	15.047 (10.984-19.138)	0.306	0.580
Odynophagia	25.856 (15.952-35.761)	37.651 (27.034-48.268)	2.448	0.118
General symptom items				
Trouble swallowing saliva	46.620 (38.851-54.389)	39.195 (2.477-75.913)	1.322	0.250
Choking when swallowing	30.160 (37.746-56.480)	40.378 (2.828-77.928)	2.702	0.100
Dry mouth	47.113 (37.746-56.480)	52.764 (23.262-82.266)	0.113	0.737
Trouble with taste	54.998 (41.515-68.481)	66.300 (-)	1.822	0.177
Coughing	31.409 (18.541-44.276)	40.378 (34.299-46.457)	3.139	0.076
Speech problems	46.620 (33.087-60.154)	66.300 (-)	5.147	0.023

Values are presented as Median (95%CI).

*P*< 0.05 indicates a statistically significant difference in the median time to deterioration between the two groups of ESCC patients.

*P*-value based on the log-rank test.

### Preoperative high DAI and AGR were positively correlated with postoperative quality of life in patients with ESCC

Using the Cox proportional hazard model to analyze the association between preoperative DAI and AGR with postoperative quality of life in patients with ESCC. In univariate analysis, preoperative high DAI-AGR is associated with postoperative improvement in emotional function (HR=0.575, 95%CI:0.368-0.898), dysphagia (HR=0.688, 95%CI:0.478-0.989), and speech problems (HR=0.525, 95%CI:0.298-0.925). After adjusting for covariates, high DAI-AGR can still improve postoperative emotional functioning (HR=0.682, 95%CI:0.492-0.983) and speech problems (HR=0.514, 95%CI:0.280-0.944) in patients with ESCC (**Table 5**; **Supplementary Table 1**).

**Table 5:** Association between preoperative DAI and AGR and EORTC QLQ-C30/EORTC QLQ-OES18 scale in ESCC patients.

Domain/scale	Univariate		Multivariate	
	*HR* (95*%CI*)	*P* value	*HR* (95*%CI*) ^*^	*P* value
QLQ-C30				
Global health status/QOL	1.240 (0.892-1.724)	0.200	1.269 (0.891-1.807)	0.187
Physical functioning	1.070 (0.773-1.480)	0.684	0.923 (0.649-1.314)	0.658
Role functioning	1.148 (0.807-1.632)	0.444	1.123 (0.763-1.652)	0.557
Emotional functioning	0.575 (0.368-0.898)	0.015	0.682 (0.429-0.983)	0.042
Cognitive functioning	0.880 (0.563-1.378)	0.578	0.894 (0.554-1.444)	0.647
Social functioning	0.863 (0.569-1.309)	0.488	1.061 (0.672-1.675)	0.799
Fatigue	0.863 (0.593-1.257)	0.444	0.820 (0.545-1.234)	0.341
Nausea/vomiting	0.960 (0.649-1.421)	0.838	1.079 (0.695-1.677)	0.734
Pain	0.803 (0.530-1.217)	0.301	0.780 (0.499-1.218)	0.274
Dyspnea	0.728 (0.474-1.116)	0.145	0.745 (0.470-1.179)	0.209
Insomnia	0.861 (0.579-1.281)	0.460	0.792 (0.512-1.225)	0.295
Appetite loss	0.763 (0.508-1.146)	0.193	0.807 (0.514-1.266)	0.350
Constipation	0.956 (0.582-1.569)	0.858	1.108 (0.650-1.886)	0.707
Diarrhea	0.955 (0.637-1.433)	0.825	0.976 (0.622-1.532)	0.915
QLQ-QES18				
Dysphagia	0.688 (0.478-0.989)	0.043	0.767 (0.519-1.134)	0.184
Eating problems	0.845 (0.577-1.237)	0.386	0.819 (0.544-1.234)	0.340
Reflux	0.909 (0.649-1.274)	0.581	0.850 (0.577-1.253)	0.412
Odynophagia	0.669 (0.445-1.098)	0.120	0.671 (0.416-1.084)	0.103
Trouble swallowing saliva	0.741 (0.444-1.237)	0.252	0.724 (0.421-1.248)	0.245
Choking when swallowing	0.680 (0.428-1.080)	0.102	0.653 (0.396-1.078)	0.096
Dry mouth	0.927 (0.595-1.445)	0.737	0.890 (0.555-1.428)	0.629
Trouble with taste	0.676 (0.381-1.199)	0.180	0.777 (0.428-1.410)	0.406
Coughing	0.654 (0.408-1.050)	0.079	0.687 (0.414-1.138	0.145
Speech problems	0.525 (0.298-0.925)	0.026	0.514 (0.280-0.944)	0.032

*Adjusting for sex, age, marital status, education level, family income per month, smoker, drinker, postoperative radio-chemotherapy, TNM stage, ALT, AST, and creatinine.

*P*-value less than 0.05 was considered significant.

*P*-value based on univariate and multivariate Cox regression analyses.

**Figure 1:**
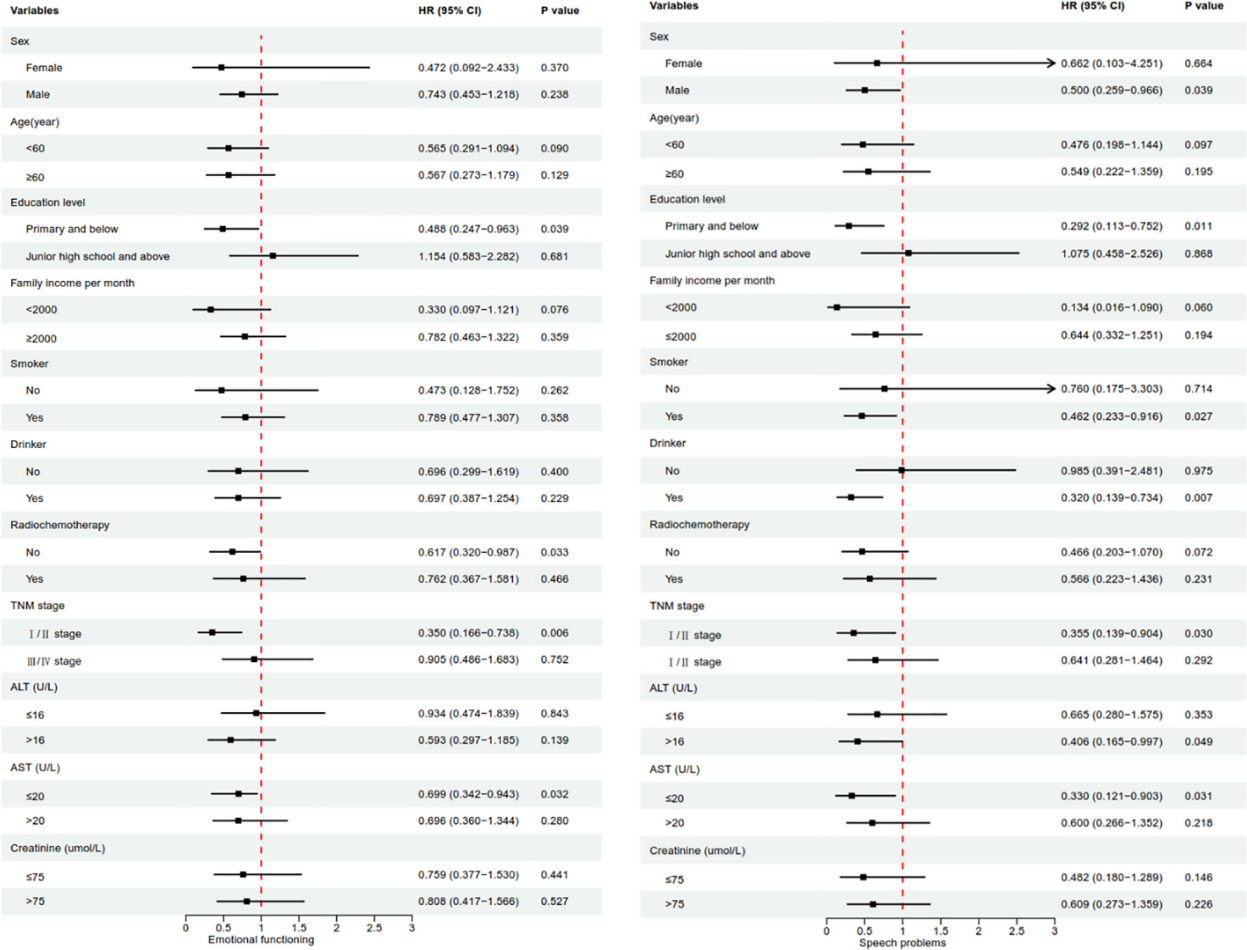
Baseline characteristics stratified analysis of forest map. *P*-value based on multivariate Cox regression analyses, adjusting for all variables except the stratification variables. *P*-value less than 0.05 was considered significant.

Further stratified by demographic and clinical characteristics, it was found that high DAI-AGR was mainly associated with postoperative emotional functioning improvement in ESCC patients at primary and below (HR=0.488, 95%CI:0.247-0.963), without radio-chemotherapy (HR=0.617, 95%CI:0.320-0.987), stage I/II (HR=0.350, 95%CI:0.166-0.738), and low levels ALT (HR=0.699, 95%CI:0.342-0.943). In terms of speech problems, the improvement effect of high DAI-AGR is more significant in males (HR=0.500, 95%CI:0.259-0.966), primary and below (HR=0.292, 95%CI:0.113-0.752), smoking (HR=0.462, 95%CI:0.233-0.916), drinking (HR=0.320, 95%CI:0.139-0.734), stage I/II (HR=0.355, 95%CI:0.139-0.904), high levels ALT (HR=0.406, 95%CI:0.165-0.997), and low levels AST (HR=0.330, 95%CI:0.121-0.903) ESCC patients (**Figure 1**). What’s more, further stratified analysis by quartiles of age revealed that the improvement effect of high DAI-AGR on emotional functioning was more pronounced in the 56~60 age group, while no significant effects were observed in other age groups. Additionally, the improvement effect of high DAI-AGR on speech difficulty was more evident in individuals aged 66 and above, with no such effect observed in other age groups (**Supplementary Table 2**).

## Discussion

In this study, our results revealed that preoperative high DAI and AGR have a positive impact on postoperative HRQOL in ESCC patients. The incidence of deterioration events in multiple dimensions of quality of life in patients with high DAI-AGR was significantly lower than that in patients with low DAI-AGR, and the deterioration time of emotional functioning, dysphagia, and speech problems was significantly delayed. The multivariate Cox-hazard regression model also validated that preoperative high DAI-AGR can improve postoperative emotional functioning and speech problems in patients with ESCC, although all baseline characteristics were adjusted, the results were still significant. Stratified by baseline characteristics, the analysis results further revealed that preoperative high DAI-AGR had a more significant effect on male patients, those with primary and below, those without radio-chemotherapy, those with smoking and drinking, and those with stage I/II. These findings suggest that patients or clinicians may consider enhancing dietary intake of antioxidants, increasing levels of albumin, and implementing proactive anti-inflammatory preoperative interventions to improve postoperative quality of life.

Earlier studies (22) have proposed a proactive nutritional approach, involving high doses of various dietary antioxidants and their derivatives. This nutritional regimen can be employed to improve the quality of life and potentially extend survival time in cancer patients who show no response to standard or experimental treatments. Our research results also support this conclusion. Dietary antioxidants can exert their impact by inhibiting the matrix metalloproteinase system (MMP), thereby suppressing tumor invasion and metastasis (23). We hypothesize that this could be a crucial mechanism contributing to the improved prognosis of ESCC patients. However, we have only conducted a cross-sectional study, and in the future, more in-depth experimental research is needed to validate our hypothesis.

A high AGR indicates elevated levels of albumin or low levels of inflammation. The established relationship between high levels of albumin or AGR and favorable prognosis in cancer patients is well-established (24). Our previous study on AGR and the quality of life in ESCC patients only found that a high AGR could improve emotional functioning (11). The association between high AGR and improved postoperative quality of life in ESCC patients is primarily attributed to the anti-inflammatory and antioxidant properties of albumin. Albumin can reduce inflammation and prevent the spread of inflammation by regulating inflammatory factors such as tumor necrosis factor-α and interleukin-6, as well as protecting endothelial function (25). At the same time, albumin acts as an important non-enzyme antioxidant, with its thiol groups capable of directly scavenging reactive oxygen species (ROS) (26). It can also reduce oxidative stress damage by binding metal ions such as copper and iron, inhibiting free radicals generated by the Fenton reaction (26). Furthermore, a higher AGR suggests a balanced level of globulin, indicating that the patient may not be in a state of excessive immune activation or immune dysfunction, thus helping to reduce infection risks and promote tissue repair (10). These anti-inflammatory and antioxidant pathways help improve postoperative quality of life for ESCC patients by reducing inflammation, protecting cells from oxidative damage, and improving immune balance. However, these mechanisms still require further clinical research and experimental validation for more thorough understanding.

With the addition of dietary antioxidant intake, we observed that both high AGR and DAI could also improve speech problems. We speculate that this is primarily associated with the anti-inflammatory effects of antioxidants. Inflammation can induce speech problems by affecting esophageal muscle tissue, leading to swallowing difficulties (27). Additionally, in the study by Bhatt et al. (28), it was suggested that excessive reactive oxygen species could trigger pro-inflammatory signaling, resulting in neurodegeneration and the onset of major depressive disorder (MDD). Adequate antioxidants were found to be effective in treating this condition, aligning with our results showing improved emotional functioning. Therefore, we can propose the hypothesis that long-term dietary antioxidant consumption may reduce systemic inflammation, subsequently increasing the albumin-to-globulin ratio, ultimately improving postoperative emotional functioning and speech problems in patients. Currently, findings suggest that the anti-inflammatory and antioxidant effects of the DAI and AGR are associated with the prognosis of various cancers (16, 29). In particular, the antioxidant role of diet has been found to be closely linked to tumor treatment outcomes (30). These results align with our findings. Our findings will also lay the foundation for future prospective studies. These insights contribute to a deeper understanding of the roles of DAI and AGR in cancer prognosis and treatment outcomes, providing a basis for further exploration and validation through more targeted research in the field.

This study also revealed an intriguing finding: compared to the non-smoking and non-drinking population, the improvement in speech problems for patients with high DAI and AGR before surgery is more pronounced among smokers and drinkers. The long-term combination of smoking and drinking has been demonstrated to be associated with an oxidative imbalance in the body (31). Therefore, the intake of dietary antioxidants may exhibit a more pronounced restorative effect on this imbalance, explaining this result. Additionally, the improvement is more evident in ESCC patients who did not undergo chemotherapy. This could be attributed to the potential impact of preoperative chemotherapy drugs on the effectiveness of dietary antioxidants and the induction of inflammation-related reactions (32), resulting in ineffectiveness in patients receiving preoperative chemotherapy. Meanwhile, we observed that the significant impact of high DAI-AGR on postoperative quality of life was primarily seen in male patients, with no significant effect observed in female patients. This may be related to gender differences in oxidative stress responses and dietary and nutritional metabolism. Men generally exhibit higher levels of oxidative stress compared to women, which could be attributed to the antioxidative effects of estrogen in females (33). Estrogen provides protective effects by regulating oxidative stress and enhancing antioxidant enzyme activity, thereby reducing oxidative damage (34). Consequently, male patients may rely more on exogenous antioxidants (e.g., dietary antioxidants) to alleviate postoperative inflammation and support tissue repair. Furthermore, during postoperative recovery, men typically require higher levels of protein and antioxidants to support their greater basal metabolic rate and tissue repair needs (35). Thus, increased levels of dietary antioxidants and AGR may have a more direct impact on improving the quality of life for male patients. In short, these differences underscore the need for tailored nutritional strategies that account for sex-specific metabolic and physiological needs, especially in recovery contexts such as post-surgery.

While our research results are promising, there are also some limitations. Firstly, we excluded individuals who did not meet the inclusion criteria, potentially leading to selection bias. Secondly, while potential biases associated with telephone follow-up and observational study design exist, we implemented measures such as standardized follow-up procedures, multiple information verifications, and confounding factor control to mitigate these biases as much as possible to a certain extent. Third, the study design did not include detailed information on patients’ chronic comorbidities during data collection, which may have overlooked their potential impact on the survey outcomes in elderly patients. However, age was included as a covariate in the model for adjustment during data analysis, and age-stratified analyses were conducted to minimize the potential influence of comorbidities as much as possible. Lastly, the dietary questionnaire relies on recalling dietary habits over the past year, and despite implementing a series of quality control measures, there may still be recall bias. At the same time, the study has notable strengths. Firstly, we utilized a composite dietary antioxidant index for an overall assessment of dietary antioxidant nutrients. Secondly, we employed the TTD model to assess quality of life, allowing for the identification of minimal clinically significant changes and facilitating early detection and intervention. Finally, by examining the combined impact of dietary and blood indicators on the quality of life in ESCC patients, our study paves the way for new treatment strategies to enhance postoperative quality of life for patients.

## Conclusions

In conclusion, our findings provide evidence that high preoperative DAI-AGR is associated with improved postoperative quality of life in ESCC patients. These results furnish essential information for patients, practitioners, and researchers, serving as valuable reference data for future studies and are expected to serve as a reference for preoperative nutritional intervention efforts.

## Data Availability

The original contributions presented in the study are included in the article/**Supplementary Material**. Further inquiries can be directed to the corresponding author.

## Supplementary material

Supplementary Table 1 Association of DAI-AGR and demographic characteristics with emotional function and speech problems.

Supplementary Table 2 Association of DAI-AGR and demographic characteristics with emotional function and speech problems after age stratification.

## References

[1] Bray F Laversanne M Sung H Ferlay J Siegel RL Soerjomataram I. Global cancer statistics 2022: GLOBOCAN estimates of incidence and mortality worldwide for 36 cancers in 185 countries. CA Cancer J Clin. (2024) 74:229–63. doi: 10.3322/caac.21834 38572751

[2] Morgan E Soerjomataram I Rumgay H Coleman HG Thrift AP Vignat J. The global landscape of esophageal squamous cell carcinoma and esophageal adenocarcinoma incidence and mortality in 2020 and projections to 2040: new estimates from GLOBOCAN 2020. Gastroenterology. (2022) 163:649–658.e2. doi: 10.1053/j.gastro.2022.05.054 35671803

[3] Baba Y Baba H Yamamoto S Shimada H Shibata T Miyazaki T. Chemotherapy-induced nausea and vomiting is less controlled at delayed phase in patients with esophageal cancer: a prospective registration study by the CINV Study Group of Japan. Dis Esophagus. (2016) 30(2):1–7. doi: 10.1111/dote.12482 27001532

[4] Haraldstad K Wahl A Andenæs R Andersen JR Andersen MH Beisland E. A systematic review of quality of life research in medicine and health sciences. Qual Life Res. (2019) 28:2641–50. doi: 10.1007/s11136-019-02214-9 PMC6761255 31187410

[5] Arraras JI Arias F Tejedor M Pruja E Marcos M Martínez E. The eortc QLQ-C30 (version 3.0) quality of life questionnaire: validation study for Spain with head and neck cancer patients. Psycho-Oncology. (2002) 11:249–56. doi: 10.1002/pon.v11:3 12112486

[6] Fujita T Okada N Sato T Mayanagi S Kanamori J Daiko H. Translation, validation of the EORTC esophageal cancer quality-of-life questionnaire for Japanese with esophageal squamous cell carcinoma: analysis in thoraco-laparoscopic esophagectomy versus open esophagectomy. Japanese J Clin Oncol. (2016) 46:615–21. doi: 10.1093/jjco/hyw040 27056967

[7] Deftereos I Kiss N Isenring E Carter VM Yeung JMC. A systematic review of the effect of preoperative nutrition support on nutritional status and treatment outcomes in upper gastrointestinal cancer resection. Eur J Surg Oncol. (2020) 46:1423–34. doi: 10.1016/j.ejso.2020.04.008 32336624

[8] Gupta D Lis CG. Pretreatment serum albumin as a predictor of cancer survival: A systematic review of the epidemiological literature. Nutr J. (2010) 9:69–85. doi: 10.1186/1475-2891-9-69 21176210 PMC3019132

[9] Ikeda S Yoshioka H Ikeo S Morita M Sone N Niwa T. Serum albumin level as a potential marker for deciding chemotherapy or best supportive care in elderly, advanced non-small cell lung cancer patients with poor performance status. BMC Cancer. (2017) 17(1):797–805. doi: 10.1186/s12885-017-3814-3 29183294 PMC5704395

[10] Li J Zhu N Wang C You L Guo W Yuan Z. Preoperative albumin-to-globulin ratio and prognostic nutritional index predict the prognosis of colorectal cancer: a retrospective study. Sci Rep. (2023) 13(1):17272–827. doi: 10.1038/s41598-023-43391-5 37828259 PMC10570287

[11] Zhang J Lin Z Zhou J Huang Y Chen S Deng Y. Effects of preoperative albumin-to-globulin ratio on overall survival and quality of life in esophageal cell squamous carcinoma patients: a prospective cohort study. BMC Cancer. (2023) 23(1):342–351. doi: 10.1186/s12885-023-10809-2 37055773 PMC10103440

[12] Reynolds J Abdel-Latif MM Babar M Kelleher D. J Cancer Res Ther. (2019) 15:185–91. doi: 10.4103/jcrt.JCRT_763_16 30880777

[13] Aiko S Kumano I Yamanaka N Tsujimoto H Takahata R Maehara T. Effects of an immuno-enhanced diet containing antioxidants in esophageal cancer surgery following neoadjuvant therapy. Dis Esophagus. (2012) 25:137–45. doi: 10.1111/j.1442-2050.2011.01221.x 21762279

[14] Bu Y Qu J Ji S Zhou J Xue M Qu J. Dietary patterns and breast cancer risk, prognosis, and quality of life: A systematic review. Front Nutr. (2023) 9. doi: 10.3389/fnut.2022.1057057 PMC9895856 36741991

[15] Dehghan P Nejati M Vahid F Almasi-Hashiani A Saleh-Ghadimi S Parsi R. The association between dietary inflammatory index, dietary antioxidant index, and mental health in adolescent girls: an analytical study. BMC Public Health. (2022) 22:1513. XXXR.H. J. doi: 10.1186/s12889-022-13879-2 35945535 PMC9361696

[16] Lv GY An L Sun XD Hu YL Sun DW. Pretreatment albumin to globulin ratio can serve as a prognostic marker in human cancers: a meta-analysis. Clin Chim Acta. (2018) 476:81–91. doi: 10.1016/j.cca.2017.11.019 29170102

[17] Nakamura J Manabe N Yamatsuji T Fujiwara Y Murao T Ayaki M. Subjective factors affecting prognosis of 469 patients with esophageal squamous cell carcinoma: a retrospective cohort study of endoscopic screening. BMC Gastroenterol. (2022) 22:319. doi: 10.1186/s12876-022-02399-3 35764928 PMC9238142

[18] Eghtesad S Hekmatdoost A Faramarzi E Homayounfar R Sharafkhah M Hakimi H. Validity and reproducibility of a food frequency questionnaire assessing food group intake in the PERSIAN Cohort Study. Front Nutr. (2023) 10. doi: 10.3389/fnut.2023.1059870 PMC10436288 37599697

[19] Anota A Savina M Bascoul-Mollevi C Bonnetain F. QoLR: an R package for the longitudinal analysis of health-related quality of life in oncology. J Stat Software. (2017) 77(12):1–30. doi: 10.18637/jss.v077.i12

[20] Musoro JZ Bottomley A Coens C Eggermont AMM King MT Cocks K. Interpreting European Organisation for Research and Treatment for Cancer Quality of life Questionnaire core 30 scores as minimally importantly different for patients with Malignant melanoma. Eur J Cancer. (2018) 104:169–81. doi: 10.1016/j.ejca.2018.09.005 30359910

[21] Wright ME. Development of a comprehensive dietary antioxidant index and application to lung cancer risk in a cohort of male smokers. Am J Epidemiol. (2004) 160:68–76. doi: 10.1093/aje/kwh173 15229119

[22] Prasad KN. Multiple dietary antioxidants enhance the efficacy of standard and experimental cancer therapies and decrease their toxicity. Integr Cancer Therapies. (2016) 3:310–22. doi: 10.1177/1534735404270936 15523102

[23] Russnes KM Möller E Wilson KM Carlsen M Blomhoff R Smeland S. Total antioxidant intake and prostate cancer in the Cancer of the Prostate in Sweden (CAPS) study. A case control study. BMC Cancer. (2016) 16:438–50. 27400803 10.1186/s12885-016-2486-8 PMC4939657

[24] Zhang F Sun P Wang ZQ Wang DS Zhang DS. Low preoperative albumin-globulin score predicts favorable survival in esophageal squamous cell carcinoma. Oncotarget. (2016) 7:30550–60. doi: 10.18632/oncotarget.v7i21 PMC5058700 27105522

[25] Zhang WJ Frei B. Albumin selectively inhibits TNF alpha-induced expression of vascular cell adhesion molecule-1 in human aortic endothelial cells. Cardiovasc Res. (2002) 55:820–9. doi: 10.1016/S0008-6363(02)00492-3 12176131

[26] Belinskaia DA Voronina PA Shmurak VI Jenkins RO Goncharov NV. Serum albumin in health and disease: esterase, antioxidant, transporting and signaling properties. Int J Mol Sci. (2021) 22(19):10318–55. doi: 10.3390/ijms221910318 34638659 PMC8508759

[27] Sonies BC. Evaluation and treatment of speech and swallowing disorders associated with myopathies. Curr Opin Rheumatol. (1997) 9:486–95. doi: 10.1097/00002281-199711000-00003 9375277

[28] Bhatt S Nagappa AN Patil CR. Role of oxidative stress in depression. Drug Discovery Today. (2020) 25:1270–6. doi: 10.1016/j.drudis.2020.05.001 32404275

[29] Chung JW Park DJ Chun SY Choi SH Lee JN Kim BS. The prognostic role of preoperative serum albumin/globulin ratio in patients with non-metastatic renal cell carcinoma undergoing partial or radical nephrectomy. Sci Rep. (2020) 10:11999. doi: 10.1038/s41598-020-68975-3 32686760 PMC7371633

[30] Muchtaridi M Az-Zahra F Wongso H Setyawati LU Novitasari D Ikram EHK. Molecular mechanism of natural food antioxidants to regulate ROS in treating cancer: A review. Antioxidants (Basel). (2024) 13(2):207–25. doi: 10.3390/antiox13020207 38397805 PMC10885946

[31] Fang XM Wang J Liu Y Zhang X Wang T Zhang HP. Combined and interactive effects of alcohol drinking and cigarette smoking on the risk of severe illness and poor clinical outcomes in patients with COVID-19: a multicentre retrospective cohort study. Public Health. (2022) 205:6–13. doi: 10.1016/j.puhe.2022.01.013 35219128 PMC8784431

[32] Rotovnik Kozjek N Kompan L Žagar T Mrevlje Ž. Influence of enteral glutamine on inflammatory and hormonal response in patients with rectal cancer during preoperative radiochemotherapy. Eur J Clin Nutr. (2017) 71:671–3. doi: 10.1038/ejcn.2017.11 28272402

[33] Martin-Millan M Castaneda S. Estrogens, osteoarthritis and inflammation. Joint Bone Spine. (2013) 80:368–73. doi: 10.1016/j.jbspin.2012.11.008 23352515

[34] Mobley JA Brueggemeier RW. Estrogen receptor-mediated regulation of oxidative stress and DNA damage in breast cancer. Carcinogenesis. (2004) 25:3–9. doi: 10.1093/carcin/bgg175 14514655

[35] Phillips SM. Dietary protein requirements and adaptive advantages in athletes. Br J Nutr. (2012) 108 Suppl 2:S158–67. doi: 10.1017/S0007114512002516 23107527

