# Supplementary material for: Association between dietary antioxidants, serum albumin/globulin ratio and quality of life in esophageal squamous cell carcinoma patients: a 7-year follow-up study
Source: Front Oncol. 2025 Jan 23;15:1428214. doi: 10.3389/fonc.2025.1428214 (PMC11798787; doi:10.3389/fonc.2025.1428214)
Supplement: Supplementary Table 1 — Association of DAI-AGR and demographic characteristics with emotional function and speech problems. [file Table1.docx]

**Supplementary Table 1**. Association of DAI-AGR and demographic characteristics with emotional function and speech problems.

| Variables | Emotional functioning | | Speech problems | |
| --- | --- | --- | --- | --- |
|  | *HR* (95*%CI*) | *P* value | *HR* (95*%CI*) | *P* value |
| Sex |  |  |  |  |
| Male | Ref (1) |  | Ref (1) |  |
| Female | 1.336 (0.555-3.218) | 0.518 | 3.746 (0.860-16.312) | 0.079 |
| Age (year) |  |  |  |  |
| <60 | Ref (1) |  | Ref (1) |  |
| ≥60 | 0.943 (0.668-1.330) | 0.738 | 1.110 (0.733-1.679) | 0.623 |
| Marital status |  |  |  |  |
| Unmarried | Ref (1) |  | Ref (1) |  |
| Married | 0.595 (0.137-2.574) | 0.487 | 0.250 (0.056-1.120) | 0.070 |
| Widowed | 0.493 (0.085-2.865) | 0.431 | 0.154 (0.020-1.163) | 0.070 |
| Education level |  |  |  |  |
| Primary and below | Ref (1) |  | Ref (1) |  |
| Junior high school and above | 0.936 (0.645-1.359) | 0.729 | 0.879 (0.560-1.379) | 0.573 |
| Family income per month |  |  |  |  |
| <2000 | Ref (1) |  | Ref (1) |  |
| ≥2000 | 0.839 (0.583-1.206) | 0.343 | 1.170 (0.764-1.793) | 0.469 |
| Smoker |  |  |  |  |
| No | Ref (1) |  | Ref (1) |  |
| Yes | 1.355 (0.606-3.027) | 0.459 | 3.294 (0.798-13.604) | 0.099 |
| Drinker |  |  |  |  |
| No | Ref (1) |  | Ref (1) |  |
| Yes | 1.211 (0.814-1.801) | 0.345 | 1.215 (0.741-1.992) | 0.441 |
| Postoperative radio-chemotherapy |  |  |  |  |
| No | Ref (1) |  | Ref (1) |  |
| Yes | 0.755 (0.520-1.097) | 0.140 | 0.941 (0.603-1.468) | 0.789 |
| TNM stage |  |  |  |  |
| I–II | Ref (1) |  | Ref (1) |  |
| III–IV | 1.580 (1.090-2.289) | 0.016 | 1.326 (0.855-2.058) | 0.208 |
| ALT (U/L) |  |  |  |  |
| ≤16 | Ref (1) |  | Ref (1) |  |
| >16 | 0.831 (0.539-1.282) | 0.403 | 0.919 (0.558-1.514) | 0.740 |
| AST (U/L) |  |  |  |  |
| ≤20 | Ref (1) |  | Ref (1) |  |
| >20 | 1.373 (0.887-2.124) | 0.155 | 1.472 (0.883-2.455) | 0.139 |
| Creatinine (μmol/L) |  |  |  |  |
| ≤75 | Ref (1) |  | Ref (1) |  |
| >75 | 0.893 (0.619-1.288) | 0.545 | 1.239 (0.8111-1.893) | 0.322 |
| DAI-AGR |  |  |  |  |
| Low | Ref (1) |  | Ref (1) |  |
| High | 0.682 (0.429-0.983) | 0.042 | 0.514 (0.280-0.944) | 0.032 |

P-value less than 0.05 was considered significant.

P-value based on multivariate Cox regression analyses.
