# Supplementary material for: Association between dietary antioxidants, serum albumin/globulin ratio and quality of life in esophageal squamous cell carcinoma patients: a 7-year follow-up study
Source: Front Oncol. 2025 Jan 23;15:1428214. doi: 10.3389/fonc.2025.1428214 (PMC11798787; doi:10.3389/fonc.2025.1428214)
Supplement: Supplementary Table 2 — Association of DAI-AGR and demographic characteristics with emotional function and speech problems after age stratification. [file Table2.docx]

**Supplementary Table 2**. Association of DAI-AGR and demographic characteristics with emotional function and speech problems after age stratification.

| Age group (year) | Emotional functioning | | Speech problems | |
| --- | --- | --- | --- | --- |
|  | *HR* (95*%CI*) | *P* value | *HR* (95*%CI*) | *P* value |
| ≤55 |  |  |  |  |
| DAI-AGR | 0.986 (0.427-2.276) | 0.974 | 0.525 (0.167-1.646) | 0.269 |
| Sex | 4.019 (0.907-17.814) | 0.067 | 2.038 (0.195-21.286) | 0.552 |
| Marital status | - | - | - | - |
| Education level | 0.863 (0.455-1.636) | 0.652 | 0.595 (0.242-1.465) | 0.259 |
| Family income per month | 0.737 (0.378-1.439) | 0.371 | 4.056 (1.319-12.473) | 0.015 |
| Smoker | 1.342 (0.366-4.922) | 0.657 | 1.874 (0.242-14.538) | 0.548 |
| Drinker | 1.563 (0.692-3.527) | 0.282 | 0.727 (0.282-1.875) | 0.509 |
| Postoperative radio-chemotherapy | 1.257 (0.535-2.953) | 0.600 | 1.168 (0.389-3.508) | 0.782 |
| TNM stage | 0.911 (0.411-2.020) | 0.819 | 0.703 (0.271-1.181) | 0.467 |
| ALT (U/L) | 0.732 (0.229-2.342) | 0.599 | 0.880 (0.243-3.194) | 0.846 |
| AST (U/L) | 1.001 (0.304-3.291) | 0.99 | 0.966 (0.274-3.406) | 0.957 |
| Creatinine (μmol/L) | 0.835 (0.369-1.888) | 0.664 | 2.124 (0.803-5.616) | 0.129 |
| 56~60 |  |  |  |  |
| DAI-AGR | 0.245 (0.077-0.772) | 0.016 | 0.823 (0.226-2.990) | 0.767 |
| Sex | 6.310 (0.639-62.283) | 0.115 | - | 0.917 |
| Marital status | 1.000 (0.464-2.155) | 0.999 | 1.934 (0.603-6.208) | 0.267 |
| Education level | 0.683 (0.258-1.807) | 0.442 | 1.650 (0.589-4.626) | 0.341 |
| Family income per month | 1.209 (0.531-2.754) | 0.651 | 0.785 (0.267-2.301) | 0.659 |
| Smoker | 5.826 (0.666-50.932) | 0.111 | - | 0.917 |
| Drinker | 1.358 (0.454-4.064) | 0.584 | 0.726 (0.161-3.273) | 0.677 |
| Postoperative radio-chemotherapy | 0.981 (0.452-2.130) | 0.962 | 0.466 (0.169-1.283) | 0.139 |
| TNM stage | 1.142 (0.487-2.676) | 0.760 | 3.159 (1.044-9.557) | 0.042 |
| ALT (U/L) | 0.834 (0.325- | 0.706 | 1.747 (0.533-5.721) | 0.357 |
| AST (U/L) | 1.240 (0.466- | 0.666 | 0.853 (0.235-3.099) | 0.810 |
| Creatinine (μmol/L) | 0.857 (0.257- | 0.802 | 1.933 (0.501-7.463) | 0.339 |
| 61~65 |  |  |  |  |
| DAI-AGR | 0.889 (0.342-2.308) | 0.809 | 1.324 (0.443-3.959) | 0.615 |
| Sex | 0.049 (0.004-0.574) | 0.016 | - | 0.932 |
| Marital status | 4.766 (0.452-50.215) | 0.194 | 0.916 (0.080-10.507) | 0.944 |
| Education level | 1.417 (0.581-3.459) | 0.444 | 1.150 (0.385-3.438) | 0.803 |
| Family income per month | 0.385 (0.155-0.960) | 0.041 | 0.466 (0.186-1.166) | 0.103 |
| Smoker | 0.106 (0.010-1.121) | 0.062 | - | 0.935 |
| Drinker | 0.829 (0.340-2.022) | 0.680 | 0.947 (0.320-2.803) | 0.921 |
| Postoperative radio-chemotherapy | 0.286 (0.106-0.769) | 0.013 | 1.086 (0.387-3.047) | 0.875 |
| TNM stage | 6.715 (2.360-19.106) | <0.001 | 2.534 (0.922-6.965) | 0.071 |
| ALT (U/L) | 0.559 (0.236-1.324) | 0.186 | 0.641 (0.219-1.879) | 0.417 |
| AST (U/L) | 1.991 (0.790-5.015) | 0.144 | 3.147 (0.954-10.381) | 0.060 |
| Creatinine (μmol/L) | 1.268 (0.457-3.516) | 0.649 | 10.709 (2.663-43.067) | 0.001 |
| ≥66 |  |  |  |  |
| DAI-AGR | 0.369 (0.116-1.181) | 0.093 | 0.119 (0.015-0.947) | 0.044 |
| Sex | 0.212 (0.042-1.079) | 0.062 | 0.890 (0.083-9.494) | 0.923 |
| Marital status | 0.883 (0.384-2.027) | 0.768 | 0.598 (0.152-2.357) | 0.463 |
| Education level | 0.894 (0.386-2.071) | 0.793 | 1.142 (0.451-2.890) | 0.780 |
| Family income per month | 0.586 (0.278-1.236) | 0.160 | 0.767 (0.311-1.889) | 0.564 |
| Smoker | 0.246 (0.049-1.223) | 0.086 | 1.307 (0.162-10.575) | 0.802 |
| Drinker | 1.379 (0.617-3.081) | 0.433 | 2.277 (0.866-5.898) | 0.095 |
| Postoperative radio-chemotherapy | 0.595 (0.270-1.312) | 0.086 | 0.663 (0.246-1.791) | 0.418 |
| TNM stage | 2.236 (1.058-4.728) | 0.035 | 1.213 (0.524-2.811) | 0.652 |
| ALT (U/L) | 2.191 (0.704-6.824) | 0.176 | 0.952 (0.281-3.229) | 0.937 |
| AST (U/L) | 2.074 (0.784-5.486) | 0.141 | 3.296 (1.002-10.840) | 0.050 |
| Creatinine (μmol/L) | 0.366 (0.147-0.915) | 0.031 | 0.554 (0.203-1.512) | 0.249 |

P-value less than 0.05 was considered significant.

P-value based on multivariate Cox regression analyses.
